# Editorial

**Published:** 1842-02-05

**Authors:** 


THE MEDICAL EXAMINER.

PHILADELPHIA, FEBRUARY 5, 1842.

### MISCELLANEOUS INTELLIGENCE.

*Obituary.*—Died, at London, on the 2d December, aged sixty-five years, Dr. Birkbeck, founder of the Mechanics' Institutions for gratuitous lectures. At London, on the 4th December, Dr. Daniel D. Davis, aged sixty-nine years, lately professor of obstetric medicine in University College, London, and author of many standard medical works; Dr. D. was physician-accoucheur to the Duchess of Kent, at the birth of Queen Victoria.

*Ohio Lunatic Asylum.*—The third annual report of this institution represents it in excellent condition. Reports are given of many interesting cases of recovery from insanity, under the treatment of Dr. William M. Awl, superintendent of the institution, and his assistant, Dr. Samuel M. Smith.

*Medical Institution of Yale College.*—The degree of doctor in medicine has recently been conferred upon nineteen students of this institution.

*Geneva Medical College.*—This institution appears to be in an exceedingly prosperous state. The present course of lectures is attended by one hundred and fifty-six students and forty-eight physicians.

*Introductory Lecture by Professor Wright, of the Medical College of Ohio.*—A very clever production, containing much sound practical advice to the young practitioner.

